# Real-Time Obstacle Detection with YOLOv8 in a WSN Using UAV Aerial Photography

**DOI:** 10.3390/jimaging9100216

**Published:** 2023-10-10

**Authors:** Shakila Rahman, Jahid Hasan Rony, Jia Uddin, Md Abdus Samad

**Affiliations:** 1 Department of Computer Science, American International University-Bangladesh, Dhaka 1229, Bangladesh; 2 Department of Computer Science and Engineering, Dhaka University of Engineering & Technology, Gazipur 1700, Bangladesh; 3 Artificial Intelligence and Big Data Department, Woosong University, Daejeon 34606, Republic of Korea; 4 Department of Information and Communication Engineering, Yeungnam University, Gyeongsan-si 38541, Republic of Korea

**Keywords:** YOLOv8, wireless sensor networks (WSNs), obstacle detection, unmanned aerial vehicles (UAVs), UAV aerial photography

## Abstract

Nowadays, wireless sensor networks (WSNs) have a significant and long-lasting impact on numerous fields that affect all facets of our lives, including governmental, civil, and military applications. WSNs contain sensor nodes linked together via wireless communication links that need to relay data instantly or subsequently. In this paper, we focus on unmanned aerial vehicle (UAV)-aided data collection in wireless sensor networks (WSNs), where multiple UAVs collect data from a group of sensors. The UAVs may face some static or moving obstacles (e.g., buildings, trees, static or moving vehicles) in their traveling path while collecting the data. In the proposed system, the UAV starts and ends the data collection tour at the base station, and, while collecting data, it captures images and videos using the UAV aerial camera. After processing the captured aerial images and videos, UAVs are trained using a YOLOv8-based model to detect obstacles in their traveling path. The detection results show that the proposed YOLOv8 model performs better than other baseline algorithms in different scenarios—the F1 score of YOLOv8 is 96% in 200 epochs.

## 1. Introduction

Unmanned aerial vehicles (UAVs) have become a ubiquitous part of our daily lives, with their applications ranging from aerial photography to military reconnaissance [1,2]. The term UAV, also known as a drone, refers to a flying vehicle that can be remotely controlled or operated autonomously. The versatility of drones has made them an essential tool for a wide range of industries, from agriculture [3] and construction to emergency services and national security. Drones’ autonomy is one of their most important characteristics, especially for military and security purposes. Drones with autonomous piloting may be pre-programmed with precise flight paths and can fly without human control [4]. Since drones may be employed for reconnaissance and surveillance tasks in military and security operations, this function is very beneficial.

Another critical feature of drones, particularly for military and security purposes, is real-time object detection. Drones equipped with object detection technologies can identify and follow both stationary and moving objects in real-time [5]. Advanced sensors and cameras that drones may be fitted with allow them to instantly detect and monitor possible threats, giving military and security personnel vital knowledge in real time. For real-time object detection, cutting-edge machine learning techniques and computer vision technologies are employed. These algorithms enable drones to recognize and classify diverse things within their field of view based on patterns and characteristics discovered by their sensors and cameras. The algorithms may also be trained to identify new objects and improve their accuracy over time, making them better and better at recognizing and tracking objects in a range of situations.

However, there are a number of challenges associated with real-time object detection, particularly in the processing and interpretation of data. Drones generate a lot of data, including images, video recordings, and sensor readings, which must be instantly evaluated in order to successfully detect objects. This necessitates efficient infrastructure for data transmission and storage in addition to reliable processing hardware and software. Assuring the precision and dependability of object detection algorithms is another difficulty, particularly in complex and dynamic situations. For example, a drone flying in a crowded urban area may encounter a wide range of objects and obstacles, including buildings, trees, bikes, cars, pedestrians, animals, and other drones. The algorithms must be able to distinguish between different objects and accurately track their movements, even in challenging conditions such as poor lighting or inclement weather conditions. Despite these difficulties, real-time object identification is a critical capability of drones in a variety of applications, from aerial photography to military surveillance and beyond. Drones will probably become much more adaptable and efficient as technology develops over time, with the capacity to find and follow objects in surroundings that are more complicated and dynamic.

Alongside military and security applications, drones are also very useful for a wide range of commercial and civilian applications. For example, drones can be used for aerial photography and videography. This allows photographers and filmmakers to capture stunning images and footage from unique perspectives without risking human life [6]. Drones can also be used for mapping and surveying applications [7], allowing for the rapid and accurate collection of geographical data. In the agriculture industry, drones are increasingly being used for crop monitoring and management purposes. Drones equipped with advanced sensors and cameras can capture high-resolution images and data that can be used to detect crop stress, nutrient deficiencies, and other issues that can impact crop health and yield effects. This information can then be used to make data-driven decisions about the crop management sector, including irrigation, fertilization, and pest control. Drones are being utilized in emergency services for search and rescue operations, enabling rescuers to swiftly find and reach people in distant or inaccessible places. Drones using thermal imaging cameras can also be used to find people who went missing or who are buried under rubble or other materials [8,9].

The study in [10] improved obstacle detection and collision avoidance in drones with weight constraints; the proposed method employs lightweight monocular cameras for depth computation. It extracts key point features using computer vision algorithms like Harris corner detector and SIFT, then matches them using Brute Force Matching (BFM) to detect increasing obstacle sizes as the drone approaches. Another paper [11] presented an indoor obstacle detection algorithm that combines YOLO with a light field camera, offering a simpler setup than RGB-D sensors. It accurately calculates obstacle size and position using object information and depth maps, demonstrating higher detection accuracy indoors.

Overall, drones are a versatile and useful tool for a wide range of applications, from military and security to commercial and civilian. Drones are projected to become increasingly important in the years to come, delivering valuable insights and information in a variety of businesses and fields because of their capacity to function independently and detect objects in real time. Drones will surely advance and be able to carry out a larger variety of jobs and applications as technology continues to advance. However, it is also important to ensure that drones are used ethically and responsibly, particularly in military and security applications, to prevent potential risks and negative consequences. With the right guidance and regulations, drones have the potential to revolutionize many different industries and provide numerous benefits to society. From these perspectives, we have shown here an advanced deep learning model to effectively detect both static and moving objects in real time from unmanned aerial vehicle data. In this case, the proposed scenario is a UAV-based wireless sensor network (WSN) system where multiple UAVs collect data from a group of sensors while avoiding all obstacles in their traveling path and where the obstacles are detected based on the UAVs’ captured photography. The main contributions of this paper are as follows:

- We have introduced an obstacle detection framework in UAV-aided WSNs based on UAV aerial photography;
- First, the UAV-captured photography (i.e., images and videos) is processed using different image-processing tools;
- Second, based on the processed data, the UAVs are trained using a YOLOv8-based object detection model to detect obstacles in their traveling path;
- Finally, the results of the proposed YOLOv8-based model are analyzed for different scenarios and compared with the results of the other baseline models. The evaluation results show that the proposed YOLOv8 performs better in all cases.

This paper follows a specific organization that begins with a literature review in Section 2, which provides a background on previous research in the field. Section 3 represents the detailed system model of the proposed work. Section 4 presents the proposed methodology, outlining the steps taken to achieve the research objectives. Section 5 offers a detailed analysis of the experimental results, providing insights into the effectiveness of the proposed methodology, and, finally, Section 6 concludes the paper.

## 2. Literature Review

UAVs are currently a trending topic in the research world, with numerous studies and projects exploring their potential applications and capabilities. Using the drone camera feature to detect and classify objects is one of the most important and useful features of UAVs. Different kinds of traditional machine learning, deep learning, as well as transfer learning models have been applied and tested. For example, in [12], the authors propose three algorithms that combine an object detector and visual tracker for single-object detection and tracking in videos, using limited training data based on the convolutional neural network (CNN) model. The algorithms efficiently incorporate the temporal and contextual information of the target object by switching between the detector and tracker or using them in combination. Another study has used the TensorFlow object detection API to implement object detection on drone videos and compare the performance of different target detection algorithms [13]. CNNs with transfer learning are used to recognize objects such as buildings, cars, trees, and people. The study shows that the choice of target detection algorithm affects detection accuracy and performance consumption in different ways. Hybrid models have also shown promises; for example, [14] proposed a method for vehicle detection in UAV videos using a combination of optical flow, connected graph theory, and CNN–SVM algorithms, which achieved 98% accuracy in detecting vehicles in moving videos. However, those traditional models are computationally expensive and require a lot of processing power.

Apart from these, various lightweight models such as MobileNet, SSD, and YOLO have gathered attention for object detection using drones. Widodo et al. discussed the development of object detection using deep learning for drone-based medical aid delivery, using a combination of Single Shot Detector (SSD) and the MobileNet framework to efficiently detect and locate objects in video streams [15]. Then, a real-time drone detection algorithm based on modified YOLOv3 with improvements in network structure and multi-scale detection achieved 95.60% accuracy and 96% average precision in detecting small drone objects [16]. A paper [17] applied the YOLOv3 algorithm for robust obstacle detection. It introduced the YOLOv3 network structure, multi-scale target detection principles, and implementation steps. Experimental results demonstrated improved robustness compared to YOLOv2, with increased detection in complex backgrounds, better lighting, low illumination contrast, and even in poor lighting conditions. YOLOv3 outperforms YOLOv2 by accurately detecting small, distant targets, such as pedestrians, in front of the track. After that, the newer version, YOLOv4, was introduced, and different studies found better accuracy and speed using this version [18]. A further improved YOLOv4 model was proposed for small object detection in surveillance drones, achieving 2% better mean average precision (mAP) results on the VisDrone dataset while maintaining the same speed as the original YOLOv4 [19]. A study [20] proposed a real-time obstacle detection method for coal mine environments, addressing low illumination, motion blur, and other challenges. It combined DeblurGANv2 for image deblurring, a modified YOLOv4 with MobileNetv2 for faster detection, and SANet attention modules for better accuracy. Experimental results showed significant improvements in detection performance. However, YOLOv5 is one of the most popular methods. As an example, a study achieved high recall and mAP scores on a combined dataset from a challenge and a publicly available UAV dataset [21]. The model leverages PANet neck and mosaic augmentation to improve the detection of small objects in complex background and lighting conditions. Some other papers proposed an improvised version of the YOLOv5 model for object detection and compared its performance to the original YOLOv5 model [22,23]. Another study proposed a YOLOv5-like architecture with ConvMixers and an additional prediction head for object detection using UAVs, which were trained and tested on the VisDrone 2021 dataset. Furthermore, the authors in [24] modified the YOLOv5 architecture for better aerial image analysis performance, concentrating on uses like mapping land usage and environmental monitoring.

Then, YOLOv6, a newer version with faster inference and accuracy, came into play. A study evaluated YOLOv6 for fire detection in Korea, showing high performance and real-time capabilities [25]. XGBoost achieved the highest accuracy for object identification. Data augmentation and Efficient Net improved YOLOv6’s performance. However, limitations included smoke classification and accuracy in poorly illuminated environments. Another piece of research suggested a transfer learning-based model using YOLOv6 for real-time object detection in embedded environments [26]. Pruning and finetuning algorithms improve accuracy and speed. The model identifies objects and provides voice output. It outperforms other models, is simple to set up, and has the potential for assisting visually impaired individuals in an IoT environment. But the model faces difficulty identifying objects against textured backgrounds and potential challenges in compressing network width.

Still, the YOLOv6 model requires a large dataset and is computationally expensive. Thus, recently, YOLOv7 was introduced, producing a higher mAP on the COCO dataset than YOLOv6, using the EfficientRep backbone and SimOTA training. It is also faster and more efficient at using GPU hardware. A few studies have been conducted to perform object detection using YOLOv7 [27,28,29]. The authors in [27] used an improved YOLOv7-based object detection method designed specifically for marine unmanned aerial vehicle (UAV) photos. In [28], the proposed YOLOv7–UAV algorithm performs UAV-based object detection tasks with better accuracy, making it appropriate for use in remote sensing, agriculture, and surveillance. The improvements improve detection accuracy by altering the anchor box aspect ratios and using multi-scale training techniques. The study in [29] supports sustainable agricultural practices and presents YOLOv7 as a useful tool for automated weed detection and crop management. But, as a complex model, YOLOv7 has scope to improve accuracy and reduce noise sensitivity. Furthermore, the work in [30] was optimized for aerial photography taken by unmanned aerial vehicles (UAVs), wherein multi-scale characteristics were added to YOLOv7 to improve object detection precision in UAV photos. This is why, most recently, YOLOv8 has been revealed. In this paper, we have studied the latest version of YOLO, YOLOv8, for real-time object (i.e., obstacle) detection in wireless sensor networks (WSNs) based on unmanned aerial vehicle (UAV) photography.

## 3. System Model

**Figure 1:**
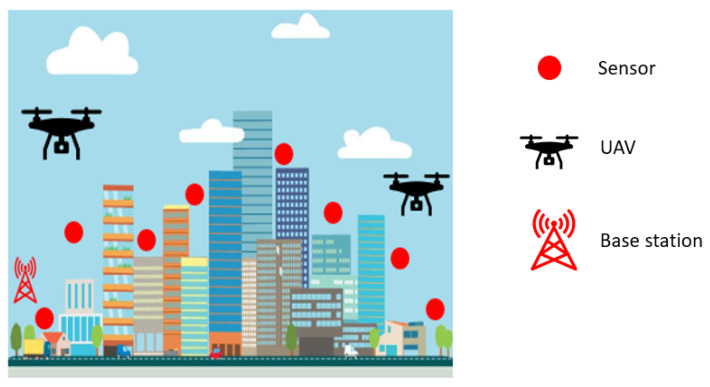
System model.

Figure 1 represents the proposed system model of the considered WSN, which includes one base station, sensor nodes, UAVs, and obstacles (i.e., buildings, trees, vehicles, etc.). The sensors are located in pre-determined positions and are randomly distributed within an area. Each sensor periodically generates data and stores them in its buffer until a UAV arrives to collect the data while capturing images and videos. The UAV-captured images and videos are used to train the UAVs to detect obstacles in their tour. Each UAV has a specific velocity and flight duration. These parameters are crucial for ensuring that the UAV can successfully collect data from all the assigned sensors and return to the base station. The battery of a UAV is assumed to be fully charged at the beginning of the tour. Then, after finishing one data collection tour, the UAV battery is recharged from the base station using a wireless method.

Moreover, let us assume that there are a set of obstacles in the environment and that the positions of these obstacles are unknown. When a UAV starts traveling to collect data, it starts capturing images and videos. Based on these aerial photographs, the UAVs are trained to detect obstacles. If a UAV detects an obstacle along its path, which means it is located between two sensors that the UAV needs to visit sequentially for data collection, the UAV must take a detour to bypass the obstacle and reach the next sensor on its intended path. This detour is required to guarantee that the UAV, despite the presence of any obstacle, may effectively travel through the environment and gather data from all the sensors. The main focus of this study is detecting the obstacles on the UAV data collection tour. On the other hand, the UAV detouring problem can be solved using the GA w/VRAOA algorithm [31] to build a UAV detour traveling path while detecting the obstacles. In general, the UAV travels to collect data from sensor nodes and the UAV data collection process is wireless, with 2 s UAV data reception time [31]. The UAV travels in the communication range of the clustered area (contained within the sensor nodes and obstacles). The clustering can be predefined or can be performed by applying the K-means algorithm, similar to [31]. Alternatively, the UAV communication security can be ensured using the efficient, secure communication infrastructure for UAVs [32].

## 4. Methodology

The following sub-sections present the details of the implementation of the proposed method.

### 4.1. Image Acquisition

**Figure 2:**
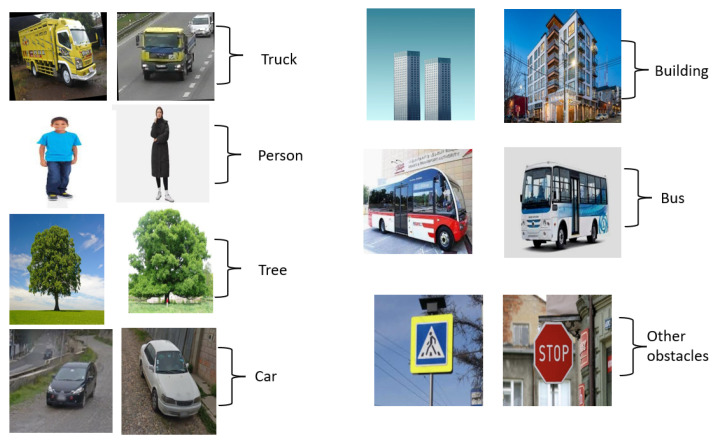
Sample dataset.

Data collection is an important step that involves gathering information from diverse, valid sources. In the context of this scenario, in this study, the data are initially collected from different online sources, including both images and videos. These online sources can be websites, social media platforms, online databases, or any other relevant online repository. A procedure for extracting the frames from the videos is used to transform the video content into photos. As a result, it is possible to compile a number of unique pictures that represent various scenes from that video. To supplement the dataset, other photographs from the internet are also collected in this dataset. Once the necessary data, including images and video frames, are collected from online sources, a custom dataset is formed. Table 1 shows the statistics of the image dataset, where we have considered seven different types of obstacles (i.e., truck, person, tree, car, building, bus, and other obstacle) for the proposed system. A dataset serves as one of the most valuable for training machine learning models, conducting research, or performing analysis. The custom dataset is curated based on the specific requirements and objectives of the project, ensuring that it encompasses a diverse range of relevant data to support the intended goals. Figure 2 shows some sample image datasets.

### 4.2. Image Pre-Processing

Data augmentation: Data augmentation is a widely used approach that helps to increase the diversity and variability of a dataset, with the goal of improving the performance and robustness of ML/AI models. During the process of collecting our real data, data augmentation techniques were applied to a few of the images to make the dataset more realistic. We have applied different data augmentation for the images, as follows.

In case 1, for dark images, a random brightness adjustment was applied as a data augmentation step. The brightness was adjusted by a percentage of −25% to +25%. This technique helped us to enhance the visibility and clarity of dark images and to make them more suitable for analysis as well as model training. In case 2, we applied the rotation augmentation technique. In this method, a random rotation is applied to the specific images as a data augmentation step. Here, the rotation angle was randomly selected between −15 and +15 degrees. The dataset becomes more similar to the real-world situations by rotating the photos, where things can appear in a variety of orientations. This specific augmentation technique enables the model to learn and generalize better, even when faced with rotated images during inference or testing.

**Figure 3:**
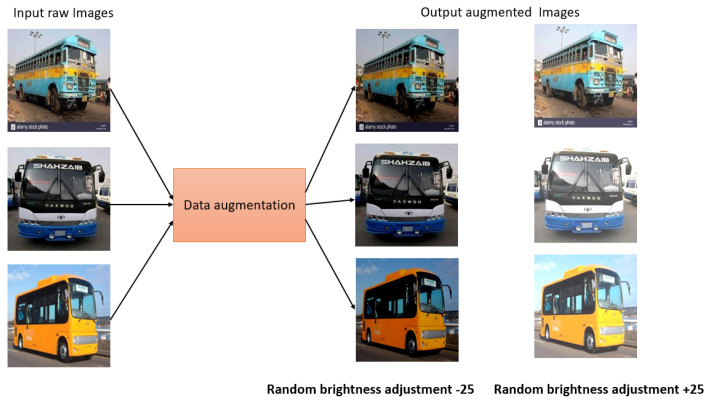
Sample data augmentation case 1.

**Figure 4:**
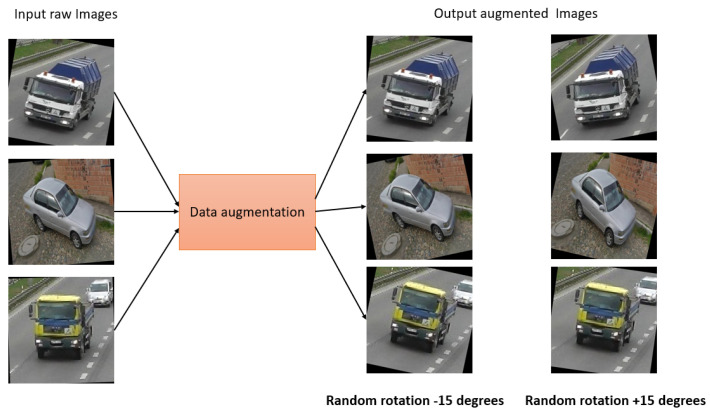
Sample data augmentation case 2.

Then, the augmented data were combined with the other raw data acquired to create a custom dataset. This custom dataset then comprised both the original raw data and the augmented data. As a result, it became more representative of real-world scenarios and encompassed a broader range of variations, enhancing the dataset’s quality and increasing the model’s ability to generalize well. The augmented data introduced additional examples with altered attributes, such as brightness-adjusted or rotated images, which helps mitigate biases. In addition, this also helps to improve the robustness of the trained model. The custom dataset serves as a valuable resource for various applications and gave us the scope to leverage a more extensive and diverse collection of data for specific tasks. This facilitates better model training, validation, and evaluation, ultimately leading to more accurate and reliable results in the targeted domain. Figure 3 and Figure 4 show the sample image augmentation for both case 1 and case 2.

### 4.3. Image Resizing and Labeling

**Figure 5:**
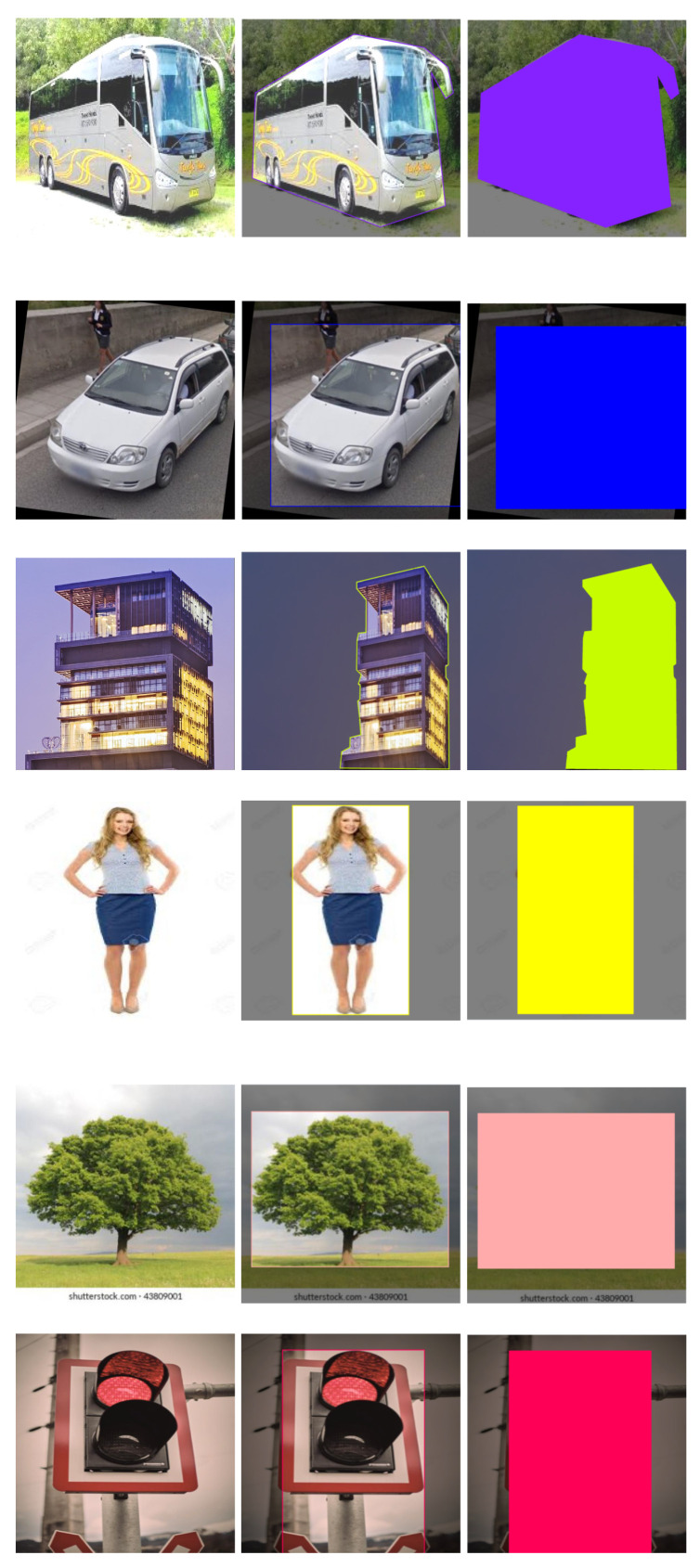
Sample labeled data.

Resizing an image means giving all of the images the same shape. Thus, the custom dataset with all images was resized to 800 × 600 pixels [33,34]. After that, the resized images were labeled according to the seven classes shown in Table 1. The Roboflow annotation operation was applied for labeling data into multiple classes. In Figure 5, we have shown some sample labeled images for each class. Here, in first column, the input images are shown, then in second column the class with a bounded box is shown, and, lastly, the bounded box layer for each class is shown with different colors in last column. The class ID in a bounding box is the name of the obstacle. For example, in this work, the seven class IDs are ‘Truck’, ‘Person’, ‘Bus’, ‘Car’, ‘Tree’, ‘Building’, and ‘Other obstacles’, as displayed in Figure 5. In this case, each bounding box indicates the obstacle, and each class ID represents the type of obstacle.

**Figure 6:**
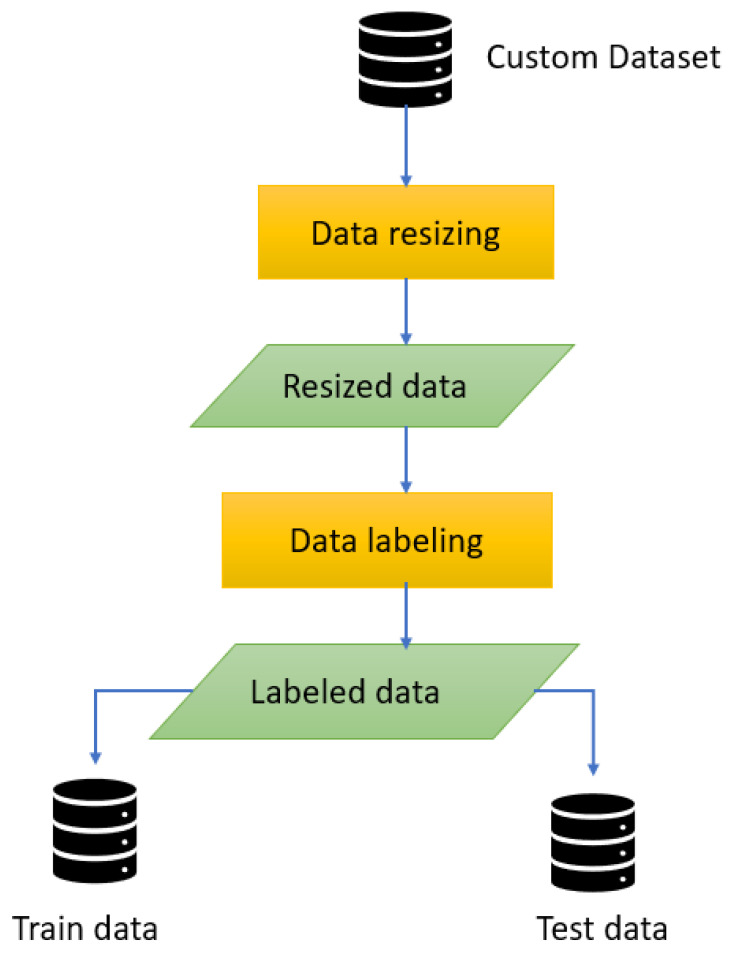
Data pre-processing steps.

All the data pre-processing steps are displayed precisely in Figure 6. After completing all the data pre-processing steps, the processed data were split into 70% train data, 20% test data, and 10% validation data for training the model.

### 4.4. Proposed Obstacle Detection Framework

**Figure 7:**
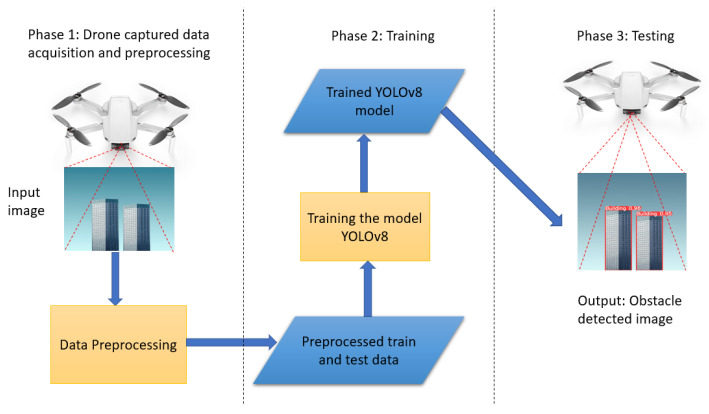
Proposed obstacle detection framework.

The proposed obstacle detection framework consists of the three main phases shown in Figure 7. In Phase 1, the system collects image or video data through the drone camera and applies pre-processing steps such as stabilization, noise reduction, and enhancement to enhance the image’s quality and usability. In Phase 2, the pre-processed data are processed to generate training and testing datasets. To provide a sufficiently diverse and representative dataset, this step encompasses activities including bounding box generation, object annotation, and data augmentation approaches. Then, the proposed YOLOv8-based model is trained using the generated processed dataset.

Finally, in Phase 3, the trained YOLOv8 model is deployed and tested for real-time object detection using the drone. The model processes the live video feed from the drone and predicts the presence and location of objects of interest in real-time. This phase enables the detection of objects while the drone is in operation, allowing for immediate response or decision-making based on the detected objects in real-time. Overall, this framework provides a systematic approach for capturing data through a drone, pre-processing the data, training an object detection model, and performing real-time object detection using the drone.

## 5. Experimental Results

This section presents an experimental evaluation of the proposed model, along with a comparison with other state-of-art models.

### 5.1. Hyperparameters

Several training settings and hyperparameters are involved in the YOLOv8-based model training for obstacle detection in the UAV data collection scheme. This section provides an overview of the hyperparameters used in the training process. Table 2 represents the names of the parameters used and their respective values. The epoch count is 200 in the training step, where the optimizer and pre-trained model are used as the stochastic gradient descent (SGD) optimizer and COCO pre-trained model, respectively. An early stopping step is implemented to prevent overfitting and to optimize the training process in the model. The training is stopped early when no improvement has been observed in the last 50 epochs. The early stopping technique with a patience value of 50 is used, which indicates that, if no improvement has been observed for 50 consecutive epochs, the training will stop automatically. Future experiments may explore adjusting the patience value for further optimization.

Furthermore, other parameters, such as batch size, learning rate, and weight decay values of 16, 0.01, and 0.001, respectively, are considered for better model optimization.

### 5.2. Model Evaluation

The model evaluation exhibited promising performance in the trained YOLOv8 model for the proposed obstacle detection in a UAV-aided data collection system. Table 3 represents the model parameters. The model evaluation was implemented on a Python platform with CUDA 12.0 and NVIDIA-SMI 525.85.12, utilizing a GTX 1650 GPU with 4 GB of graphics memory and 8 GB of RAM. The model, consisting of 225 layers and 11,138,309 parameters, demonstrated efficient computation, achieving a GFLOPs value of 28.7. The evaluation process encompassed various metrics to assess the model’s effectiveness in detecting obstacles.

### 5.3. Analysis of Results

**Figure 8:**
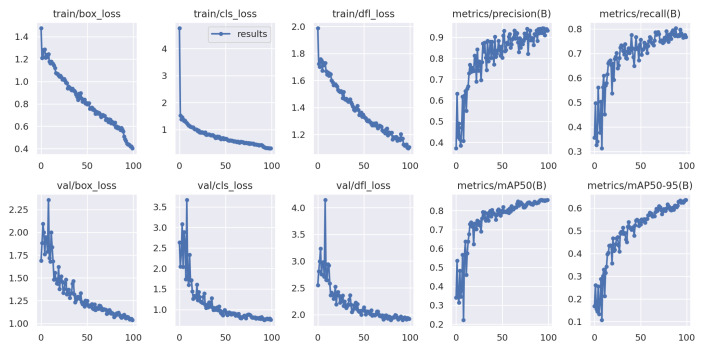
YOLOv8-based training graph with 100 epochs.

**Figure 9:**
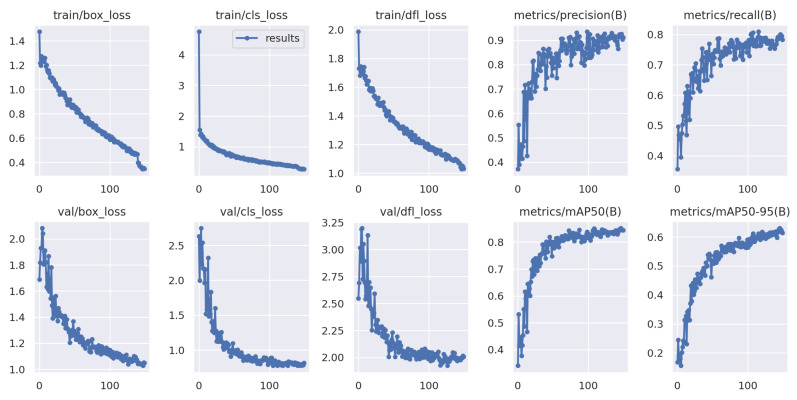
YOLOv8-based training graph with 150 epochs.

The Yolov8-based training graphs with (a) 100 epochs and (b) 150 epochs are depicted in Figure 8 and Figure 9, showing that the best results are obtained at training step 111 for the proposed scheme. Thus, the decision to train for 150 epochs is based on the observed performance, the early stopping mechanism, and the best results found in 111 iterations. Further, the proposed YOLOv8 exhibits a recall of 78.2%, a precision value of 89.99%, and a mAP of 87.4% when trained with 150 epochs.

The testing performance of the proposed obstacle detection based on the YOLOv8 model is compared with the other existing object detection models, i.e., YOLOv7 [28], YOLOv5 [24], SSD [35], and Faster-RCNN [34]. The performance is analyzed for all classes with different iteration steps with our own datasets, as shown in Table 4. As the detector learns on the validation set, the mAP@0.5 (average mean average precision) is tracked during the training phase to evaluate performance, where a greater value denotes better learning. Furthermore, the F1score is calculated from the equation

(1) F1score=(2×Precision×Recall)/(Precision+Recall)

In all cases, the YOLOv8-based model shows better accuracy, both in the F1score and mAP@0.5, which are 96% and 89%, respectively. Additionally, the model complexities for all the models are shown in Table 4, where the YOLOv7 used the highest number of trainable parameters among all models, which leads to lower generalization capacity.

**Figure 10:**
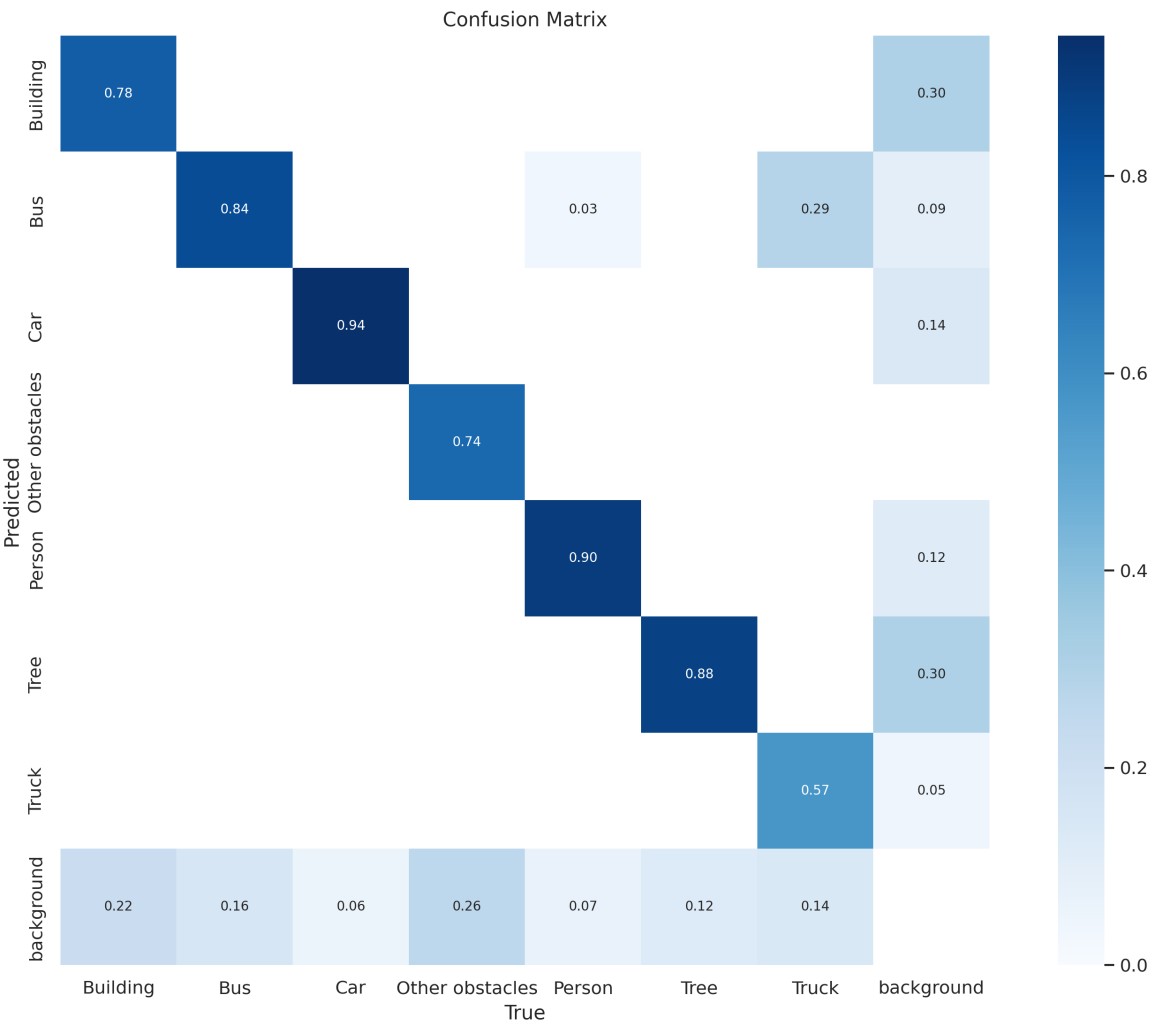
Confusion matrix diagram for 200 epochs.

Moreover, Figure 10 depicts the confusion matrix diagram at 200 iterations, where the prediction accuracy is quite high. The confusion matrix is divided into columns and rows, with each column representing a predicted class and each row representing an actual class. The prediction accuracy is highest for the car class, which is 94%, and the lowest predicted accuracy is obtained for the truck class, which is 57%. Thus, the YOLOv8-based model proved its efficacy with the 200 iteration steps, showcasing its potential for real-time obstacle detection in wireless sensor networks (WSNs) aided by UAVs in rigorous testing and analysis.

### 5.4. Visualization

**Figure 11:**
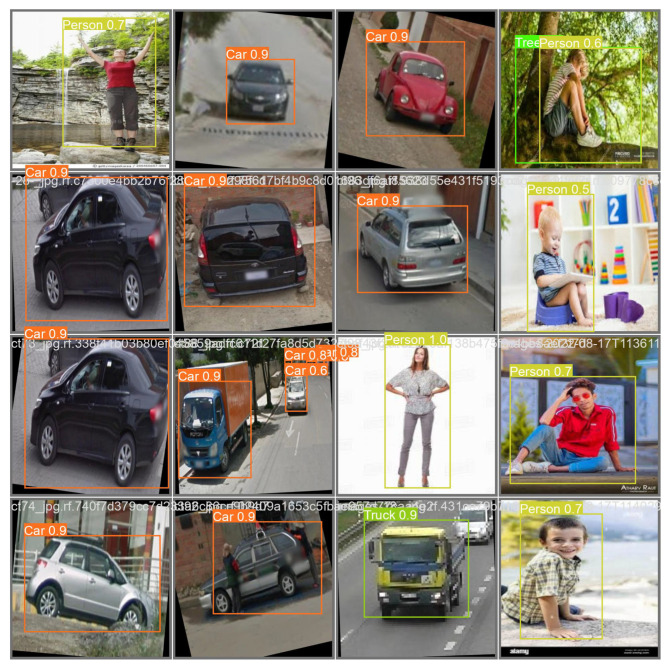
Testing performance for random data.

**Figure 12:**
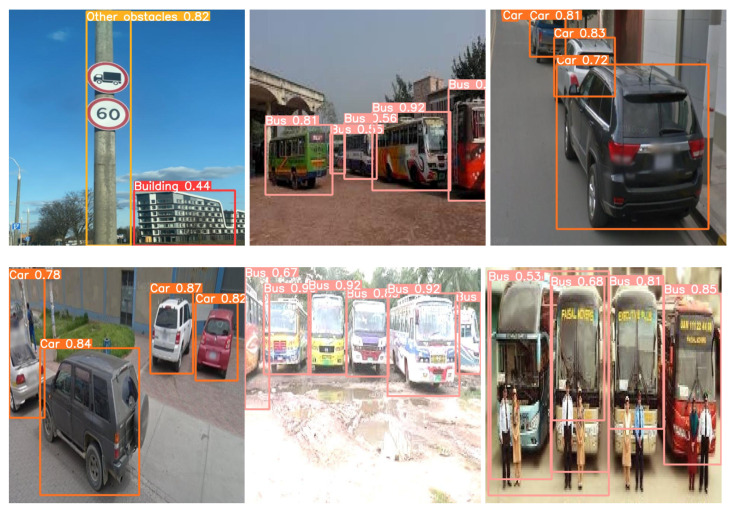
Examples of multiple detected images in one frame using the YOLOv8-based model.

The model training was conducted for a total of 200 epochs. Each epoch represents a complete iteration through the entire training dataset. The training process involves updating the model’s parameters based on the calculated loss and gradients. The model stops at 161 epochs because the best results were found in 111 steps. Thus, the same results repeat for 50 steps, as we have considered the patience parameters to be 50, and, at 161 iterations, the training is stopped. The model training process is completed in approximately 1.843 h. The duration may vary depending on the computational resources and hardware used for training. Some randomly detected obstacles are shown in Figure 11, where training steps are stopped at 161 iterations and the detection accuracy is highest. Furthermore, Figure 12 shows some sample detected images based on the proposed YOLOv8 model for multiple obstacles in one frame.

**Figure 13:**
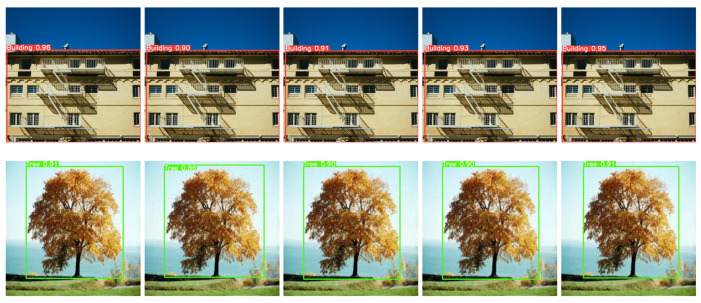

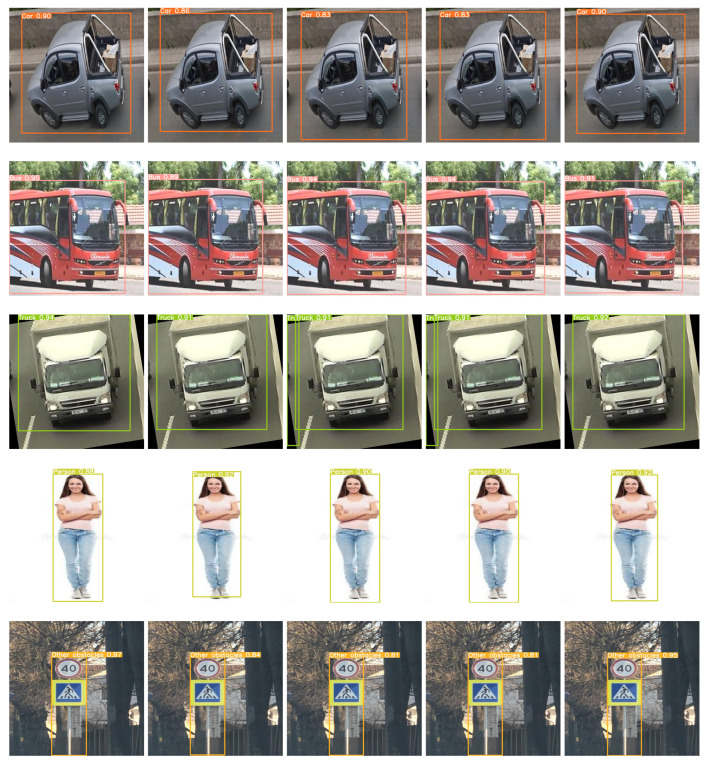
Sample detected images for YOLOv8, YOLOv7, YOLOv5, SSD, and Faster-RCNN, respectively.

The individual detection accuracies for YOLOv8, YOLOv7, YOLOv5, SSD, and Faster-RCNN in 200 training steps are shown, respectively, in Figure 13, considering all the classes. In the case of the building class, Figure 13 shows that the detection accuracies are 96%, 90%, 91%, 93%, and 95% for the YOLOv8, YOLOv7, YOLOv5, SSD, and Faster-RCNN models, respectively. Similarly, for the tree class, the detection accuracies are 91%, 86%, 90%, 90%, and 91%. For the car class, they are 90%, 86%, 83%, 83%, and 90%. For the bus class, they are 95%, 89%, 94%, 94%, and 91%. For truck, they are 94%,91%, 91%, 91%, and 92%. For person, they are 88%, 92%, 90%, 90%, and 92%. Finally, for other obstacles, they are 97%, 84%, 81%, 81%, and 95%, for the YOLOv8, YOLOv7, YOLOv5, SSD, and Faster-RCNN models, respectively. Thus, for all classes, in most cases, the detection accuracy is higher in the proposed YOLOv8-based obstacle detection model compared to the other YOLOv7, YOLOv5, SSD, and Faster-RCNN models with 200 epochs.

## 6. Conclusions

In this study, a YOLOv8-based obstacle detection framework is proposed for a UAV-aided data collection scheme in a WSN environment. This work focused on obstacle detection based on aerial photography from UAVs. The system includes multiple UAVs, multiple groups of sensors, and numerous obstacles, wherein UAVs collect data from a group of sensors while avoiding all obstacles in their path. The UAVs captured images or videos while they were traveling to collect data from the sensors. Then, the UAV aerial images and videos are processed using image processing tools, i.e., data augmentation, resizing, and labeling. After that, based on the processed data, the UAVs are trained using the YOLOv8-based model to detect obstacles in their traveling tour for both 100 and 150 iterations, as shown in Figure 8 and Figure 9. The best results are found after 111 iterations, with recall value of 78.2%, precision value of 89.99%, and 87.4% mAP. On the other hand, the predicted value in the confusion matrix shown in Figure 10 is quite high for all type of obstacles. The evaluation results show that YOLOv8 performs better than YOLOv7, YOLOv5, SSD, and Faster-RCNN in different scenarios, and the detection accuracy is also higher in YOLOv8 for different sample test images, as shown in Figure 13.

Seven types of obstacles are considered in this work. In a real-world scenario, when a UAV collects data in WSNs, the obstacles can be many other types than these seven types, which is a limitation of this work. Generally, this project intends to train a UAV to detect obstacles in its traveling path and make the UAV operation more efficient. In future work, we intend to train the UAV with bigger datasets. We also intend to solve the problem of avoiding obstacles on its tour along with the obstacle detection problem using a machine learning-based (i.e., a reinforcement learning-based) UAV path-planning approach.

## Figures and Tables

**Table 1:** Dataset statistics.

Types of Obstacles	Quantity within the Dataset	Total Data
Truck	254	
Person	166	
Tree	221	
Car	260	1376
Building	126	
Bus	247	
Other obstacles	102	

**Table 2:** Parameters used for the YOLOv8-based object detection model.

Parameters	Value
Batch size	16
Number of epochs	200
Optimizer	SGD
Pre-trained	COCO model
Learning rate	0.01
Weight decay	0.001
Patience	50

**Table 3:** Model parameters.

Parameters	Value
Model layers	225
Model parameters	11,138,309
Gradients	11,138,293
GFLOPs	28.7

**Table 4:** Testing performance of YOLOv8 with YOLOv7, YOLOv5, SSD, and Faster-RCNN.

Model	Epoch	Class	Trainable Parameters	F1score	mAP@0.5
Proposed YOLOv8	100	All	25.9M	0.92	0.75
Proposed YOLOv8	200	All	25.9M	0.96	0.89
YOLOv7 [28]	100	All	36.9M	0.87	0.72
YOLOv7 [28]	200	All	36.9M	0.89	0.78
YOLOv5 [24]	100	All	21.2M	0.91	0.81
YOLOv5 [24]	200	All	21.2M	0.92	0.85
SSD [35]	100	All	13M	0.81	0.86
SSD [35]	200	All	13M	0.84	0.89
Faster-RCNN [34]	100	All	19M	0.88	0.85
Faster-RCNN [34]	200	All	19M	0.90	0.88

## Data Availability

The dataset is available online: https://github.com/shakilaBD/YOLOv8_obstacle_detection (accessed on 5 October 2023).
